# Isolation and Molecular Identification of Nipah Virus from Pigs

**DOI:** 10.3201/eid1012.040452

**Published:** 2004-12

**Authors:** Sazaly AbuBakar, Li-Yen Chang, A.R. Mohd Ali, S.H. Sharifah, Khatijah Yusoff, Zulkeflie Zamrod

**Affiliations:** * University of Malaya, Kuala Lumpur, Malaysia;; † Veterinary Research Institute, Ipoh, Perak, Malaysia;; ‡ Universiti Putra Malaysia, Selangor, Malaysia;; § Universiti Kebangsaan Malaysia, Selangor, Malaysia

**Keywords:** Malaysia, Nipah virus, Pigs, dispatch

## Abstract

Nipah viruses from pigs from a Malaysian 1998 outbreak were isolated and sequenced. At least two different Nipah virus strains, including a previously unreported strain, were identified. The findings highlight the possibility that the Malaysia outbreaks had two origins of Nipah virus infections.

An outbreak of febrile encephalitis with high death rates (≈56%) occurred among pig farmers in a pig-farming community in Tambun, Perak, Malaysia, in 1998 (*1*). The disease spread southward within 4 to 6 months to several other pig-farming communities near or around Seremban, a city ≈300 km from Tambun (*2*). By April 1999, at least 85 deaths were reported in Seremban; 15 were recorded in Tambun (*3*). All the cases from Tambun were recorded before the outbreak in Seremban. During the outbreaks, pigs in Seremban and Tambun manifested acute respiratory distress syndrome and encephalitis and subsequently died (*2**,**3*). Since the incidence of the disease in humans paralleled the occurrence of the disease in pigs, infected pigs were presumed to be the main and perhaps only source of infections in humans (*4**,**5*). Culling almost one million suspected infected pigs effectively curtailed the spread of the disease, except for a cluster of infections, the last to be reported, which occurred in the south, in Sungai Buloh, in 1999 (*3*).

Nipah virus (NV) was eventually isolated from patients manifesting the typical pig-farming–associated fatal encephalitis, confirming the viral source of the infection (*4**,**5*). The whole genome sequence of the virus was determined, and its close phylogenetic relationship to Hendra virus (HV) was shown (*6**,**7*). The viruses were designated as members of a new genus, *Henipavirus*, of the *Paramyxoviridae* family (*8**,**9*). Several other human isolates were also sequenced and found to share a high degree of sequence similarity to that of the first isolate (*10*). More recently, an NV was isolated from flying foxes of Tioman Island, located east of peninsular Malaysia (*11*). The virus had such high sequence similarity to all the human NV isolates that it was suggested as the potential source of NV (*12*). It was proposed that pigs in the north contracted the infection through contact with NV of flying foxes (from flying fox urine or leftover fruit) and that humans then acquired the infection by direct handling infected pigs (*12**–**14*). Movement of infected pigs was responsible for subsequent foci of outbreaks in the south (*2**,**3*). Whether the index outbreak in Tambun was a result of a single event (transmission of a flying fox NV to pig), which resulted in clonal propagation and transmission of the virus to pigs and subsequently humans, was unclear. To date, no reports have indicated that NV found in humans shared similar genome sequences to those found in pigs, although NV has been detected and isolated from infected pig samples (*15*). We present results from analyses of the whole genome sequence of three representative NV isolates from pigs from the three outbreak clusters, one from the north, Tambun, and two from the south, Seremban and Sungai Buloh.

## The Study

In this study, NV from pigs (NV/MY/99/VRI-0626, NV/MY/99/VRI-1413, and NV/MY/99/VRI-2794) were isolated by the Veterinary Research Institute, Malaysia. The isolate from a human patient sample from Seremban (NV/MY/99/UM-0128) was isolated at the Department of Medical Microbiology, Faculty of Medicine, University of Malaya, Malaysia. NV of pigs was isolated after infection of Vero (African green monkey kidney) cells with lung tissue samples of pigs that died from the acute respiratory distress syndrome and encephalitis. In Vero cell cultures, all the NV pig isolates manifested similar cytopathologic effects. RNA was extracted from NV-infected cells at passage three, and the genome of the four virus isolates was sequenced in its entirety and analyzed together with the genome sequences of all other NV isolates available in GenBank (Table). The genome sequence of the human NV isolate AF212302, designated as CDC, was used as the reference sequence. The sequences were aligned and manually edited. The phylogenetic trees were constructed and displayed as previously described (*16*). All new NV genome sequences were deposited in European Molecular Biology Laboratory (accession numbers AJ564621, AJ564622, AJ564623, and AJ627196).

A high sequence similarity (>99%) between NV sequences of pigs and humans was noted after aligning the whole genome sequences. A maximum likelihood phylogenetic tree, constructed by using pig NV genome sequences, showed that NV isolates of pigs clustered tightly together with all known human and flying fox Nipah viruses (Figure A). As expected, all NV sequences clustered with HV to form a distinct group from other established genera within the *Paramyxovirinae* subfamily. The NV-Tambun showed the most divergent genome sequence from all other known Nipah viruses, in a phylogram constructed by using cumulative nucleotide sequence differences (Figure B). The overall divergence value was nonetheless small (<1%); hence, the isolate remained within the NV cluster.

**Figure:**
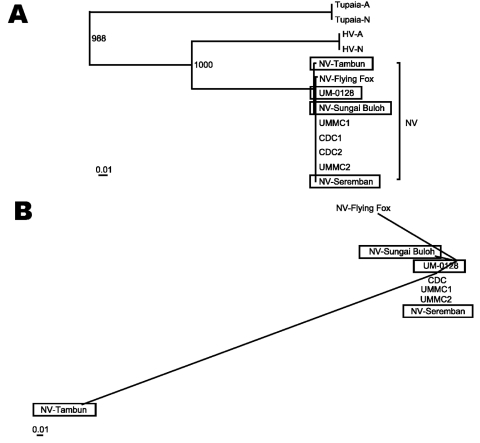
Phylogenetic trees illustrating the relationships of the pig Nipah virus isolates to all other known Nipah viruses and the related members of subfamily *Paramyxovirinae*. A) The maximum likelihood tree was drawn by using alignments of the full genome sequences. All the new isolates described in the study are shown in boxes. Abbreviations used and accession numbers not described elsewhere in the text are in parenthesis: Tupaia paramyxovirus (Tupaia-A) (AF079780); (Tupaia-N) (NC_002199), and Hendra virus (HV-A) (AF017149); (HV-N) (NC_001906). B) Unrooted maximum likelihood plot was constructed by using alignments of all the nucleotide differences in the Nipah virus gene coding regions (N, P, M, F, and G) shown in Table A1, Table A2, and Table A3 by artificially treating all the differences as a single stretch of nucleotide sequence.

Subsequent analysis of the deduced amino acid sequences of the NV-Seremban pig isolate showed that they were identical to the human NV isolates CDC and UMMC2 (Table A1, Table A2, Table A3). By contrast, the NV-Sungai Buloh pig isolate–deduced amino acid sequences were identical to the human NV isolates UMMC1 and UM-0128. NV-Seremban differed from NV-Sungai Buloh at only one amino acid position (1645) within the polymerase protein (L). Both isolates, however, differed from the flying fox NV isolate, NV-Flying Fox, at three amino acid positions, residues 30, 206, and 348 in the coding regions of nucleoprotein (N), phosphoprotein (P), and fusion protein (F), respectively. In contrast, the NV-Tambun pig isolate had a distinct signature sequence in comparison to all other NV. NV-Tambun differed from all known NV at 47 nucleotide positions; 28 of these differences occurred within the virus coding regions. The nucleotide differences were translated into amino acid changes at 11 positions; residues 274, 304, and 378 of the P protein, residues 147 and 250 of the matrix protein (M) and F protein, respectively, residues 20 and 272 of the glycoprotein (G), and residues 223, 1645, 1753, and 2039 of the L protein. Amino acid changes noted in the highly phosphorylated P protein at positions 274 and 304 resulted in residue changes from serine to arginine and threonine to alanine, respectively. These changes may reduce the potential phosphorylation sites in the P protein since serine, threonine, tyrosine, and histidine residues are the common targets for protein phosphorylation. Substitution of amino acids at positions 223 (threonine → asparagine), 1645 (serine → phenylalanine) and 2039 (histidine → asparagine) in L protein may also reduce the number of predicted potential phosphorylation sites on L. A substitution of amino acid isoleucine for asparagine at position 20 of the G protein added a potential glycosylation site apart from the eight identified N-linked glycosylation sites (*6*). However, the additional potential glycosylation site is located at the cytoplasmic tail of the protein and might make the addition of an additional glycan extremely unlikely, if not impossible. No nucleotide and consequently deduced amino acid variations, however, were observed in the N protein.

## Conclusions

Findings reported here present for the first time molecular evidence that at least two major strains of NV of pigs were circulating during the 1998 NV outbreak in Malaysia, one strain from the initial outbreak in the north (NV-Tambun) and the other strain from the subsequent outbreak approximately 4 months later in the south (NV-Seremban and NV-Sungai Buloh). The NV-Seremban and NV-Sungai Buloh pig isolates had identical sequences to those reported from human infections, which confirmed that the infections in humans during the southern outbreak originated from infected pigs. No record of isolation of the NV-Tambun is available from patients from the initial outbreak in Tambun or from the subsequent outbreaks. Isolation of NV-Seremban and NV-Sungai Buloh was not reported from the Tambun outbreak. Hence, ascertaining if the two major strains originated from the same initial focus of infection, Tambun, is not possible. Alternatively, the NV-Tambun could be the basal ancestral strain from which the later southern strain evolved. Two findings suggested this hypothesis: the Tambun outbreak occurred at least 4 months before the Seremban outbreak, and the sequence differences between NV-Seremban and NV-Sungai Buloh occurred as a result of genetic drift, a phenomenon not uncommon amongst RNA viruses. Then again, this occurrence is unlikely considering that the genome sequence of NV-Tambun diverges from the NV-Flying Fox, purportedly the initial source of NV infections. In addition, given that the NV sequences of both humans and pigs (UM-0128, NV-Seremban and NV-Sungai Buloh), sequenced independently in different laboratories, were practically identical, the sequence differences were not likely caused by inherent polymerase chain reaction errors or adaptation to tissue culture conditions. Therefore, the NV-Tambun strain is the more likely causal agent for the initial outbreak among pigs in Tambun, resulting from an infection acquired from a yet-to-be-identified source. By contrast, the subsequent outbreaks in the south were due to the pig NV isolates with higher sequence similarities to the NV-Flying Fox of Tioman Island. This finding implied that the 1998 Malaysia NV outbreak is unlikely to be due to a single transmission of NV from flying foxes of Tioman Island to pigs, but it points to the possibility of at least two different origins of NV infections.

## Appendix

**Table A1:** Comparisons of the genome sequences of Nipah virus isolates^a^

Gene:	N	P						M			F						G						L															
NV isolate	Nucleotide position																																					
	89	617	820	828	910	1132	2124	312	439	495	523	567	749	951	1017	1043	59	264	306	450	543	814	9	117	153	668	2226	2541	2704	3657	4714	4749	4934	5257	5268	6115	6486	6609
CDC	c	c	a	c	a	g	t	a	a	t	c	g	c	g	c	t	t	g	c	g	a	a	t	g	g	c	g	c	c	a	c	c	c	a	c	c	a	g
UMMC1	.	.	.	.	.	.	.	.	.	.	.	.	.	.	.	.	.	.	.	.	.	.	.	.	.	.	.	.	.	.	.	.	t	.	.	.	.	.
UMMC2	.	.	.	.	.	.	.	.	.	.	.	.	.	.	.	.	.	.	.	.	.	.	.	.	.	.	.	.	.	.	.	t	.	.	.	.	.	.
NV-Flying Fox	t	t	.	.	.	.	.	.	.	.	.	.	.	.	.	c	.	.	t	a	.	.	-	-	-	-	-	-	-	-	-	-	-	-	-	-	-	-
UM-0128	.	.	.	.	.	.	.	.	.	.	.	.	.	.	.	.	.	.	.	a	.	.	.	.	.	.	.	.	.	.	t	.	t	.	.	.	.	.
NV-Tambun	.	.	c	t	g	a	c	g	g	c	t	a	t	a	.	.	a	a	.	.	g	g	c	a	.	a	a	t	.	g	.	.	t	g	t	a	g	a
NV-Seremban	.	.	.	.	.	.	.	.	.	.	.	.	.	.	.	.	.	.	.	.	.	.	.	.	.	.	.	.	t	.	.	.	.	.	.	.	.	.
NV-Sungai Buloh	.	.	.	.	.	.	.	.	.	.	.	.	.	.	t	.	.	.	.	a	.	.	.	.	.	.	.	.	.	.	.	.	t	.	.	.	.	.

^a^Results shown are a summary of all the nucleotides (a) and its corresponding deduced amino acids (b) differences in the NV gene coding regions and nucleotides differences in the NV noncoding regions (c) of the NV-Tambun, NV-Seremban, NV-Sungai Buloh, and UM-0128 in comparison to other known NV isolates.

**Table A2:** . Comparisons of the genome sequences of Nipah virus isolates^a^

Gene:	N	P						M			F						G						L															
NV isolate	Amino acid position																																					
	30	206	274	276	304	378	708	104	147	165	175	189	250	317	339	348	20	88	102	150	181	272	3	39	51	223	742	847	902	1219	1572	1583	1645	1753	1756	2039	2162	2203
CDC	T	P	S	G	T	E	N	Q	S	L	L	K	T	L	I	M	I	Q	G	L	L	T	D	Q	R	T	L	P	L	P	L	H	S	M	D	H	K	K
UMMC1	.	.	.	.	.	.	.	.	.	.	.	.	.	.	.	.	.	.	.	.	.	.	.	.	.	.	.	.	.	.	.	.	F	.	.	.	.	.
UMMC2	.	.	.	.	.	.	.	.	.	.	.	.	.	.	.	.	.	.	.	.	.	.	.	.	.	.	.	.	.	.	.	.	.	.	.	.	.	.
NV-Flying Fox	I	L	.	.	.	.	.	.	.	.	.	.	.	.	.	T	.	.	.	.	.	.	-	-	-	-	-	-	-	-	-	-	-	-	-	-	-	-
UM-0128	.	.	.	.	.	.	.	.	.	.	.	.	.	.	.	.	.	.	.	.	.	.	.	.	.	.	.	.	.	.	.	.	F	.	.	.	.	.
NV-Tambun	.	.	R	.	A	K	.	.	G	.	.	.	I	.	.	.	N	.	.	.	.	A	.	.	.	N	.	.	.	.	.	.	F	V	.	N	.	.
NV-Seremban	.	.	.	.	.	.	.	.	.	.	.	.	.	.	.	.	.	.	.	.	.	.	.	.	.	.	.	.	.	.	.	.	.	.	.	.	.	.
NV-Sungai Buloh	.	.	.	.	.	.	.	.	.	.	.	.	.	.	.	.	.	.	.	.	.	.	.	.	.	.	.	.	.	.	.	.	F	.	.	.	.	.

^a^Results shown are a summary of all the nucleotides (a) and its corresponding deduced amino acids (b) differences in the NV gene coding regions and nucleotides differences in the NV noncoding regions (c) of the NV-Tambun, NV-Seremban, NV-Sungai Buloh, and UM-0128 in comparison to other known NV isolates.

**Table A3:** . Comparisons of the genome sequences of Nipah virus isolates^a^

Genome:	Nucleotide position																					
NV isolate	201	295	520	529	1147	1669	1780	2035	2097	2215	2381	4952	6241	6461	8847	8864	8872	11124	11309	11315	18173	18180
CDC	c	t	a	c	g	a	g	a	c	g	c	t	t	a	a	t	g	t	a	g	t	t
UMMC1	.	.	.	.	.	.	.	.	.	.	.	.	.	.	.	.	.	.	.	.	.	.
UMMC2	.	.	.	.	.	.	.	.	.	.	.	.	.	.	.	.	.	.	.	a	.	.
NV-Flying Fox	t	.	.	.	.	.	.	.	.	.	t	.	.	.	.	.	.	.	-	-	-	-
UM-0128	.	.	.	.	.	.	.	.	.	.	.	.	.	.	.	.	.	.	.	.	.	.
NV-Tambun	.	c	g	a	a	g	t	c	a	a	.	a	c	c	g	c	a	c	t	.	c	g
NV-Seremban	.	.	.	.	.	.	.	.	.	.	.	.	.	.	.	.	.	.	.	.	.	.
NV-Sungai Buloh	.	.	.	.	.	.	.	.	.	.	.	.	.	.	.	.	.	.	.	.	.	.

^a^Results shown are a summary of all the nucleotides (a) and its corresponding deduced amino acids (b) differences in the NV gene coding regions and nucleotides differences in the NV noncoding regions (c) of the NV-Tambun, NV-Seremban, NV-Sungai Buloh, and UM-0128 in comparison to other known NV isolates.

**Table:** Nipah virus isolates used in the study

Isolate	Host	Accession no.
CDC	Human	AF212302
UMMC1	Human	AY029767
UMMC2	Human	AY029768
NV-Flying Fox	Flying foxes	AF376747
UM-0128 (NV/MY/99/UM-0128)^a^	Human	AJ564623
NV-Tambun (NV/MY/99/VRI-0626)^a^	Pig	AJ627196
NV-Seremban (NV/MY/99/VRI-1413)^a^	Pig	AJ564622
NV-Sungai Buloh (NV/MY/99/VRI-2794)^a^	Pig	AJ564621

^a^New isolates described in the study.
